# Gastroprotective Effect of Quercus infectoria Olivier Galls on Ethanol-Induced Gastritis in Rats

**DOI:** 10.7759/cureus.56459

**Published:** 2024-03-19

**Authors:** Heba M Eltahir

**Affiliations:** 1 Department of Pharmacology and Toxicology (Biochemistry Division), Taibah University, Medina, SAU

**Keywords:** gastric submucosa, gastritis, gastroprotective, total acidity, quercus infectoria galls

## Abstract

One of the common inflammatory disorders that substantially affects the stomach and its mucosa is gastritis. It can be induced by non-steroidal anti-inflammatory drugs (NSAIDs), antibiotics, alcohol, *Helicobacter pylori* infection, and stress. These factors affect cellular regeneration, mucus production, and bicarbonate secretion, resulting finally in inflammation and ulceration. Ethanol-induced gastritis is one of the commonly used models for studying the pathology of gastritis and investigating the effect of drugs in managing the disease. Several drugs, such as proton pump inhibitors (PPIs), are available to control and correct the pathological signs of gastritis; however, the side effects of such drugs represent an obstacle to their applications in many cases. *Quercus infectoria* (QI) Olivier galls are formed as a pathological response to wasp insults to the tree. They are rich in several bioactive molecules, e.g., gallotannins that have been shown to be effective in several inflammatory conditions due to their antioxidant and anti-inflammatory potentials. In this study, we aimed to evaluate the therapeutic potential of QI gall extract (QIGE) in treating ethanol-induced gastritis in rats. To test this, 20 adult male Swiss rats were divided into four groups: healthy control, ethanol-treated (80% in water, 5 ml/kg, per oral gavage), ethanol + omeprazole (20 mg/kg, per oral gavage), and ethanol + QIGE (300 mg/kg, per oral gavage). QIGE was administered for seven days before ethanol administration, which took place three hours after the last QIGE dose. Three hours after ethanol intake, animals were euthanized, gastric content was collected, and stomach tissue was examined for macroscopic changes and then fixed to be further utilized for histological assessment by hematoxylin and eosin (H&E), periodic acid-Schiff (PAS), and Masson’s trichrome staining. Ethanol treatment significantly decreased gastric pH and increased gastric acidity compared to healthy control. It also induced clear morphological and histological damage and ulceration, depleted mucus on the gastric epithelium, and induced edema and collagen deposition in gastric submucosa. The QIGE treatment ameliorated the changes in gastric pH and total acidity. It also protected stomach tissue from ethanol-induced ulceration, histopathological changes, edema, and collagen deposition. The protective effects of QIGE were comparable to those of omeprazole. In conclusion, QI gall extract possesses a promising gastroprotective effect against ethanol-induced gastritis.

## Introduction

Gastritis is an inflammatory disorder that affects the mucosal membranes of the stomach and can be induced by a wide variety of factors that may include drugs, such as non-steroidal anti-inflammatory drugs (NSAIDs) and antibiotics, excessive alcohol intake, *Helicobacter pylori*, and viral infections, in addition to stress. These factors negatively impact the protective barrier lining the stomach by affecting cellular regeneration, mucus production, and bicarbonate secretion, in addition to abolishing the antioxidant capacity of gastric tissues [1]. Although gastritis can be either acute or chronic, in both cases, symptoms range from abdominal pain, nausea, and vomiting, taking into account that chronic gastritis may be asymptomatic in some individuals. Disease symptoms sound tolerable, but untreated gastritis may develop into gastric ulcers (GUs), bleeding, and an increased risk of carcinogenesis in the stomach [2,3].

One of the commonly utilized experimental models of gastritis that mimic the human form of the disease is induced by ethanol ingestion in rodents [4]. Several studies have attributed alcohol-induced gastric lesions to increased oxidative stress as a result of accumulating reactive oxygen and nitrogen species (ROS/NOS). These reactive molecules initiate a cascade of molecular events due to their ability to oxidize lipids and protein in addition to their deleterious effects on DNA, resulting in alteration of cell permeability and activation and recruitment of macrophages. These changes activate the signaling pathways of nuclear factor kappa-B (NFκB) and its downstream pro-inflammatory cytokines, e.g., interleukin-6 (IL-6) and tumor necrosis factor-α (TNF-α), which augment and propagate the inflammatory response [4].

Activated macrophages also produce and release variable molecules that aid the inflammatory response, such as prostaglandin E2 (PGE2) and nitric oxide (NO). While PGE2 is utilized in producing various cytokines and chemo-attractant molecules, NO alters vasculature permeability and aids edema at the site of inflammation [5]. It is to be noted that the reported effect of ethanol to increase secretion of gastric juice along with its inflammation-promoting effect initiates a pro-apoptotic event on gastric mucosal cells and abrogates the protective effect of nuclear factor erythroid-2 (Nrf-2) and hemoxygenase-1 (HO-1) [6].

Based on the findings that oxidative stress and inflammation are major players in the pathogenesis of gastritis, several natural products that possess antioxidant and anti-inflammatory properties have been investigated for their potential protective and therapeutic effect against ethanol-induced gastritis, such as rosmarinic acid, red ginseng, curcumin, and resveratrol, and various polyphenolic compounds [7-9].

*Quercus infectoria *Olivier (an oak tree belonging to the *Fagaceae* (*Quercaceae*) family) has been shown to contain various bioactive compounds in every plant part including pathological *Quercus Infectoria* galls (QI galls), which are formed in response to wasps laying their eggs into the tree. Based on previously published phytochemical analysis, these galls have been reported to contain a high percentage of gallotannins (up to 70%), gallic acid, syringic acid, ellagic acid, B-sitosterol, essential oils, and carbohydrates [10].

Long ago, QI gall powder and extracts were effectively used in traditional medicine to treat a wide range of ailments, such as tonsillitis, dental- and gum problems, hemorrhage, and parasitic infections due to QI gall effects, such as astringent, antibacterial, antifungal, antiparasitic, and antiviral, in addition to its use as antipyretic, anti-inflammatory, anti-diabetic, and anti-cancer [11]. Some of the therapeutic effects of QI galls were attributed to their inhibitory effect on nitric oxide and superoxide radicals via their free radical scavenging capacity, which is attributed to the high content of phenolic compounds [12]. QI gall extract was shown to possess its anti-inflammatory effect both in vivo and in vitro, where the effect was mediated via its inhibitory effect on the signaling pathway of NFkB, TNF-α, and IL-6 [11,13].

Despite the wide range of therapeutic effects of QI gall extract, its therapeutic potential in protecting against ethanol-induced gastritis has not yet been elucidated. Thus, in this work, we aimed to investigate the possible therapeutic effect of QI gall extract in an animal model of ethanol-induced gastritis.

## Materials and methods

Preparation of the QI gall extract

**Figure 1:**
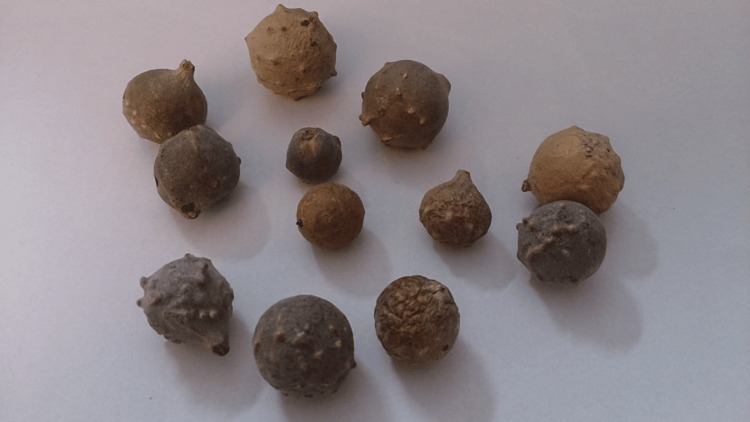
Morphology of QI gall nuts purchased from local suppliers QI: *Quercus infectoria*

QI galls were purchased from a local supplier and authenticated by members of the Pharmacognosy Department, College of Pharmacy, Taibah University (Figure 1).

After being cleaned from dust, the galls were first crushed using a mortar, then ground to fine powder, and extracted three times in 80% methanol (1:10 w/v, 24 hours at room temperature). After filtration, QI gall extract (QIGE) fractions were pooled and dried under vacuum, and the dry-, solvent-free extract was stored in airtight containers at 4˚C until needed.

Animals and experimental design

Age-mate, male Swiss albino rats (180-220 g) were utilized in the current study. Animals were purchased from the animal facility of the College of Pharmacy, Taibah University, and were kept in polypropylene animal cages at 25±2 ˚C room temperature and 12 hours of light/dark cycles. They had free access to standard animal chow and water and were allowed to acclimatize for one week before starting the experimental procedures. The study protocol was approved by the Board of the Research Ethical Committee of the College of Pharmacy, Taibah University, Medina, Kingdom of Saudi Arabia (approval no. COPTU-REC-87-20240128).

For the pharmacological experiments, animals were randomly assigned to four groups (n=5 per group) as follows: Group 1 (healthy control group), Group 2 (ethanol-treated group), Group 3 (ethanol+omeprazole-treated group (20 mg/kg, as a reference drug)), and Group 4 (ethanol + QIGE (QI gall extract)-treated group (300 mg/kg)).

Groups 1, 2, and 3 received a daily single dose of saline per oral gavage from the first day to the seventh day, whereas Group 4 received a single daily dose of QIGE dissolved in 5% carboxymethylcellulose (300 mg/kg) for the same period. All animals were fasted for 15 hours (with free access to water) before receiving the corresponding treatments on the seventh day, i.e., animals of Group 3 received a single oral dose of omeprazole (20 mg/kg in saline) and animals of Group 4 received their last dose of QIGE. Animals of Groups 1 and 2 also received their last saline dose. Three hours after the last dose of treatment on the seventh day, Groups 2, 3, and 4 received a single oral dose of 80% ethanol (5 ml/kg). After three hours of ethanol treatment, all animals were sacrificed under deep anesthesia using thiopental sodium (40 mg/kg, intraperitoneal), and their stomachs and blood were collected. The serum was isolated from blood and stored at -80 ˚C until needed.

Evaluation of gastric acidity

After collection of the stomach, the content (gastric juice) was collected from each stomach, centrifuged (3500 rpm, 15 min), and the supernatants’ pH was analyzed for acidity using a digital pH meter after diluting the volume 1:1 with distilled water [14].

Total acidity was also calculated by titration of gastric juice (1:1 diluted, after centrifugation) against 0.01N NaOH in the presence of phenolphthalein as an indicator according to a previously published method [14]. The amount of NaOH consumed was recorded for each sample and total acidity was calculated as mEq/L based on the formula:

Total acidity = volume of NaOH x normality x 100 mEq/L

Gastric ulcer score

After being collected, the stomach from each animal was cut open along the large curvature and rinsed well with ice-cold normal saline, then dried by plotting with filter paper, and pinned flat on a cardboard for investigation of macroscopic changes in the stomach tissues of the different test groups. A scoring system was utilized as an index for gastric ulcer (gastric ulcer index, GUI), where 0 indicates healthy stomach lining with no manifestations, 1 indicates red-colored lining (hyperemia) to hemorrhagic spots, 1.5-2 indicates hemorrhagic streaks to minor ulcer (five ulcers of less), 3 indicates more than five small ulcers, 4 indicates small and large ulcers, and 5 indicates a fully ulcerated stomach with perforation [15].

Histopathological examination

After being collected, opened, and thoroughly rinsed in cold normal saline, stomach tissues from the different test groups were fixed in 10% buffered paraformaldehyde (PFA) solution overnight at 4˚C. Fixed tissues were washed in phosphate-buffered saline (PBS) before being dehydrated in increasing alcohol concentration, then cleared in xylol, and embedded in paraffin. Five-micrometer-thick sections were cut, defatted, and rehydrated before being stained with hematoxylin and eosin (H&E), periodic acid-Schiff (PAS), or Masson’s trichrome stain according to the manufacturer’s instructions. Stained sections were blindly examined under a light microscope and scored by a specialized histopathologist. 

Statistical analysis

Statistical analyses performed in the current study were done using GraphPad Prism 6 (GraphPad Software Inc., CA, USA). Data presented in the study were all expressed as mean ± SEM. Analysis of variance (ANOVA) test and then the Tukey-Kramer test were utilized to analyze the data. P values <0.05 were considered as significant.

## Results

Gastric ulcer gross manifestations and ulcer scoring

**Figure 2:**
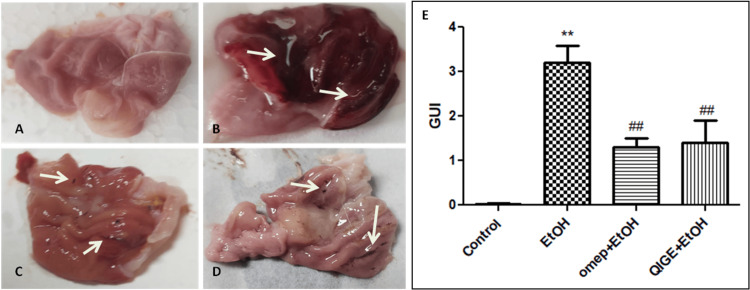
Effect of the different treatments on the gross features of inner stomach tissues A: Healthy control stomach with no abnormal discoloration or ulceration. B: Stomach from animals treated with ethanol only, showing severe hyperemia and large ulcers (arrows). C: Stomach from animals treated with omeprazole three hours before ethanol ingestion, showing almost normal features with minor petechial lesions (arrows). D: Stomach from animals treated with *Quercus infectoria* (QI) extract for seven days before induction of gastritis with ethanol, showing almost normal tissue coloration and few hemorrhagic lesions (streaks) (arrows). E: Quantitative estimation of ulcerogenic effect in the different groups, ethanol-treated group (EtOH), omeprazole + ethanol-treated group (omep+EtOH), QIGE + ethanol-treated group (QIGE+EtOH). Data are presented as mean ± SEM; n = 5 per group. * denotes a significant difference from healthy control, and # denotes a significant difference from ethanol-treated animals (p < 0.05). QIGE: *Quercus infectoria *gall extract, EtOH: ethanol, SEM: standard error of the mean, omep: omeprazole

Ethanol ingestion affected the stomach gross features of the corresponding animal group. As shown in Figure 2B, severe hyperemia with multiple hemorrhagic ulcers (arrows, Figure 2B) compared to the healthy control group (Figure 2A), showing a significant difference in the GU score between the two groups (Figure 2E). Pretreatment with omeprazole significantly protected the stomach tissue from the detrimental effect of ethanol administration with the exception of minor ulcerations, as can be observed in Figure 2C (arrows) and Figure 2E. Using QIGE as a prophylactic drug effectively and significantly protected against alcohol-induced ulceration, however, few ulcers could be still detectable in this test group compared to the healthy control (arrows, Figure 2D, 2E).

Evaluation of gastric acidity

**Figure 3:**
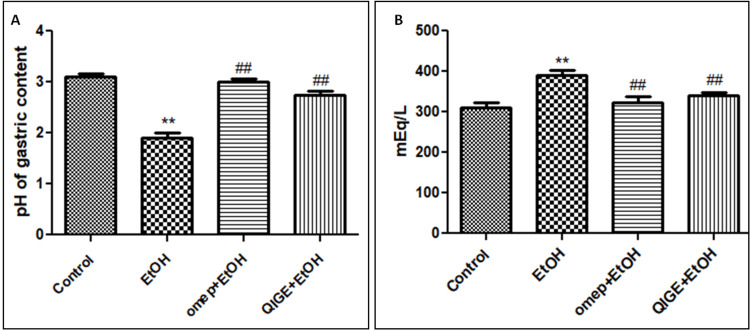
Effect of the different treatments on the pH of the gastric content and gastric total acidity Ethanol administration significantly reduced gastric pH and increased gastric acidity compared to control. Pre-treatment with QIGE or omeprazole significantly ameliorated ethanol-induced alteration in pH and gastric acidity. Ethanol-treated group (EtOH), omeprazole+ethanol-treated group (omep+EtOH), QIGE+ethanol-treated group (QIGE+EtOH). Data are presented as mean ± SEM; n = 5 per group. * denotes a significant difference from healthy control, and # denotes a significant difference from the ethanol-treated animals (p < 0.05). QIGE: *Quercus infectoria* gall extract, EtOH: ethanol, SEM: standard error of the mean, omep: omeprazole

Ethanol administration without previous protective treatment significantly decreased the pH value of the gastric contents and increased total gastric acidity compared to healthy control animals (p < 0.05; Figures 3A, 3B). By contrast, pre-treating animals with QIGE and omeprazole before administration of ethanol protected against ethanol-induced reduction in pH and increased total acidity, showing values that are significantly different compared to ethanol treatment alone (p < 0.05). It is to be noted that no significant difference could be detected when comparing gastric total acidity after pre-treatment with QIGE or omeprazole to healthy controls (p > 0.05; Figure 3B).

Histopathological examination

Effect of the Different Treatments on Histological Features

**Figure 4:**
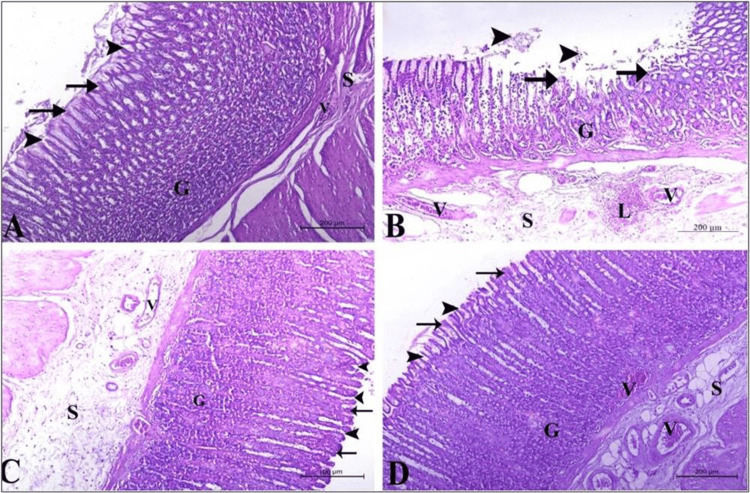
Effect of the different treatments on stomach histology (hematoxylin and eosin (H&E) staining). A: Section from the healthy control stomach showing normal gastric wall architecture with a thick layer of mucous and normal columnar epithelium in addition to a thin layer called gastric submucosa (S) containing numerous blood vessels (V). B: Section from the ethanol-treated stomach showing damaged gastric mucosa, with signs of degeneration (arrow), sloughing (arrowhead), and ulceration in the gastric epithelium and the gastric glands (G). Gastric submucosa (S) appeared very thick, enclosing highly congested blood vessels (V), and infiltrating lymphocytes (L). C: Section from the omeprazole-treated group stomach showing an almost normal gastric wall with a thick submucosal (S) layer containing congested blood vessels (V). D: Sections from QIGE-treated animals showing a normal gastric wall, gastric epithelium (arrow), gastric glands (G), and gastric submucosa (S). (H&E stain, bars = 200 µm).

By investigating H&E-stained healthy control stomach sections under a light microscope, one can observe normal architecture with a thick layer of mucous covering the gastric mucosa that shows normal columnar epithelium lining the stomach and gastric glands (G). These gastric glands appear as tubular and coiled glands lined by secretory cells, ending by shallow and narrow gastric pits (arrowhead). Underneath the gastric mucosa, a thin layer of connective tissue (submucosa, S) with numerous blood vessels (V) can be detected (Figure 4A). 

By contrast, a dramatic alteration in the stomach tissue architecture can be observed in the alcohol-treated animals in the form of degenerating gastric epithelium and the gastric glands (G, arrows), sloughing (arrowhead), and ulceration. Highly congested blood vessels (V) in an abnormally thick, edematous gastric submucosa (S) in addition to infiltrated lymphocytes (L) were also detectable in this group (Figure 4B) compared to the healthy control animals.

Interestingly, most signs of epithelial damage and inflammation observed in the ethanol-treated group were abolished in the stomach sections from the QIGE pre-treated animals, as sections show preserved gastric histological architecture that is comparable to the healthy control group with few congested blood vessels (Figure 4D). Sections from the omeprazole-treated animals looked indistinguishable from both healthy control and QIGE-treated animals regarding gastric mucosa, but a relatively thick submucosa (S) with mildly congested blood vessels could be detected in this section (Figure 4C).

Table 1 represents the quantitative analysis of various parameters that evaluate the integrity of the gastric tissue using a scoring system that was designed as follows: 0 = no pathological change; 1 = percent tissue damage < 25%; 2 = percent tissue damage 26-50%; 3 = percent tissue damage 51-75%; 4 = percent tissue damage 76-100%. Meanwhile, for the evaluation of mucus accumulation, a score of 4 represents maximum accumulation and a score of 0 represents no mucus accumulation. From the table, one can observe improved histological features when animals were pre-treated with QIGE before inducing gastritis compared to animals that were not pre-treated. 

**Table 1:** Histopathological scoring* of gastric tissue in the treated groups *Histopathological scoring of tissue injury in the gastric mucosa was scored in degrees as follows: 0 = no change; 1 = < 25% tissue damage; 2 = 26–50% tissue damage; 3 = 51–75% tissue damage; 4 = 76–100% tissue damage. For mucus accumulation, 4 represents the highest accumulation and 0 represents the least accumulation. QIGE: *Quercus infectoria *gall extract, EtOH: ethanol, omep: omeprazole

Groups	Mucous accumulation	Degeneration of gastric epithelium	Sloughing of the gastric epithelium	Ulceration of the gastric mucosa	Congestion of gastric vessels	Edema in the submucosa
Healthy control group	4	0	0	0	0	0
Ethanol-treated group	0	3	3	3	3	4
Omep+EtOH-treated group	1	2	1	1	2	2
QIGE+EtOH-treated group	3	1	0	0	2	1

Effect of the different treatments on the production of gastric mucosa glycoproteins

**Figure 5:**
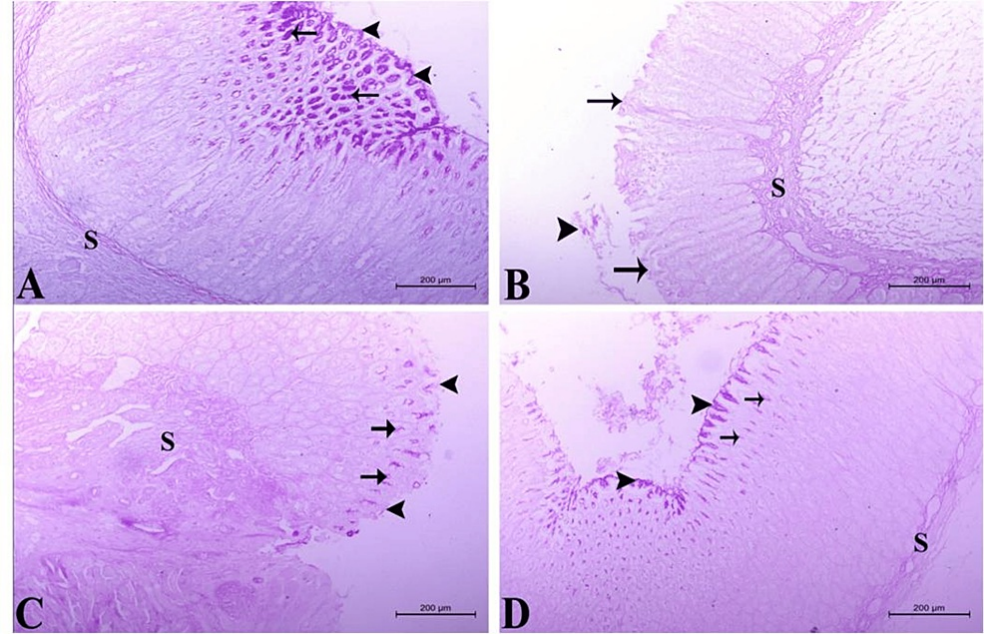
Effect of the different treatments on PAS staining of stomach glycoproteins. A: Section from the healthy control stomach showing a strong PAS reaction (arrowhead) in the mucus layer and gastric glands containing mucous secretory cells in the uppermost part (arrow). B: Section from the ethanol-treated stomach showing severely damaged gastric mucosa lacking any mucous materials (arrow) with signs of degeneration, sloughing (arrowhead), and ulceration in the gastric epithelium and glands. The gastric submucosa (S) appeared very thick. C: Section from the omeprazole-treated stomach with an almost normal gastric wall containing some PAS-positively stained gastric mucus glands with secretory cells (arrow) and covered by a very thin layer of mucous materials (arrowhead). D: Section from the QIGE-treated animals showing a strong PAS reaction (arrowhead) relative to a thick layer of mucous and multiple gastric glands containing mucous secretory cells in the upper part (arrow). PAS stain, bars = 200 µm. PAS: periodic acid–Schiff

To evaluate the integrity of the mucosal layer on the upper surface of the gastric tissue and the efficiency of glycoprotein production, sections from different test groups were stained with PAS stain that should stain mucosal cells with a bright magenta color. As observed in Figure 5A, control sections showed an intense positive staining signal in the mucus cells (arrows) and a thick mucus layer (arrowheads, Figure 5A) with healthy, mildly positive underlying submucosa (S).

Ethanol treatment of the stomach of the corresponding group induced severe loss of the mucosal lining as can be detected by the negative PAS staining signal (Figure 5B) and the thickened submucosal layer (S).

Although both the omeprazole and QIGE pre-treatment partially protected against such deleterious effect of ethanol on the mucus layer compared to the healthy control, the effect of QIGE in retrieving the mucus layer (arrowheads) and mucus glands (arrows) was much stronger compared to that of omeprazole as observed by the intensity and distribution of PAS staining (Figure 5C, 5D).

Effect of the different treatments on gastric submucosal connective tissue

**Figure 6:**
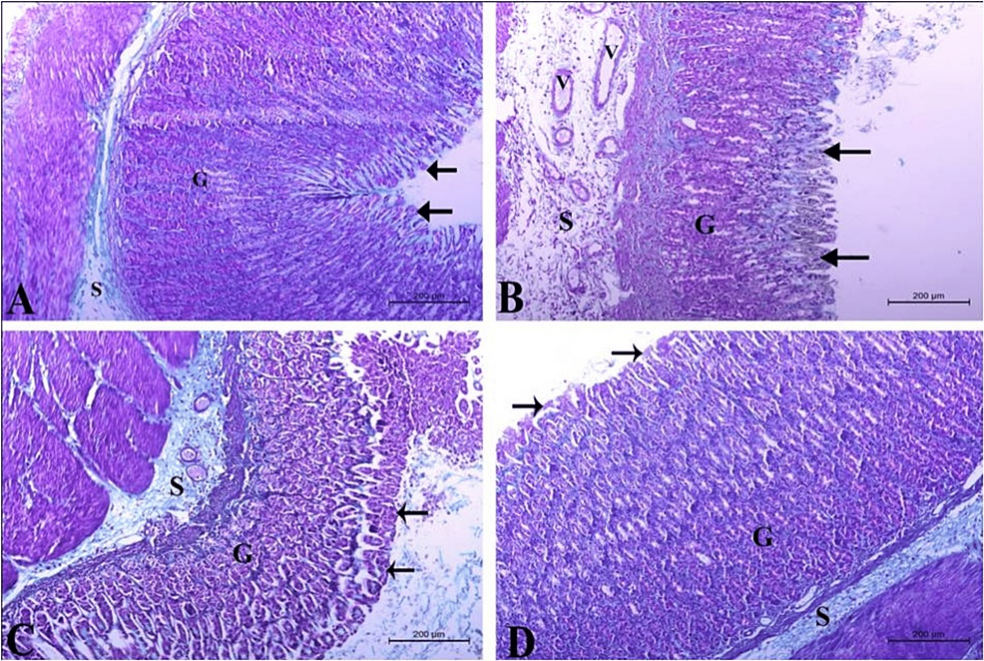
Effect of the different treatments on collagen deposition and edema. A: Section from the healthy control stomach showing a normal gastric wall with normal gastric epithelium (arrow), glands (G), and submucosa (S), with minimal deposition of collagen in the connective tissue. B: Ethanol-treated group showing massively damaged gastric epithelium (G, arrows) with signs of degeneration, sloughing, and ulceration. The gastric submucosa (S) appeared very thick with congested blood vessels (V). C: Section from the omeprazole-treated stomach with an almost normal gastric wall but showing a thick submucosal layer (S) containing congested blood vessels (V). D: Section from QIGE-treated animals with normal gastric wall and normal gastric epithelium (arrow) and gastric glands (G) in addition to gastric submucosa (S) with almost normal thickness and minimal collagen deposition. Masson’s trichrome staining, stain bars = 200 µm. QIGE: *Quercus infectoria *gall extract

In an attempt to compare the changes in the thickness of connective tissue and collagen in the submucosa after the different treatments, stomach sections were stained with Masson’s trichrome staining. As can be seen in Figure 6A, healthy control sections show a normally thin submucosal layer (S) containing few blood vessels. By contrast, sections from the ethanol-treated animals show highly thickened, positively stained submucosa (S) with multiple congested blood vessels (V) indicating edema and increased collagen deposition (Figure 6B).

Pre-treating animals with omeprazole partially ameliorated submucosal thickening and collagen deposition, as shown in Figure 6C, where a moderately thick submucosa (S) can be observed compared to submucosal thickness in ethanol-treated ones (Figure 6B). In addition, some congested blood vessels were still detectable in the submucosa of the omeprazole-treated group (V, Figure 6C). Interestingly, the pre-treatment with QIGE before induction of gastritis successfully alleviated the signs of submucosal edema as observed by normalized submucosal thickness (S) and almost lack of congested blood vessels in addition to preservation of gastric wall thickness (G, Figure 6D).

Quantitative evaluation of changes in morphological features

**Figure 7:**
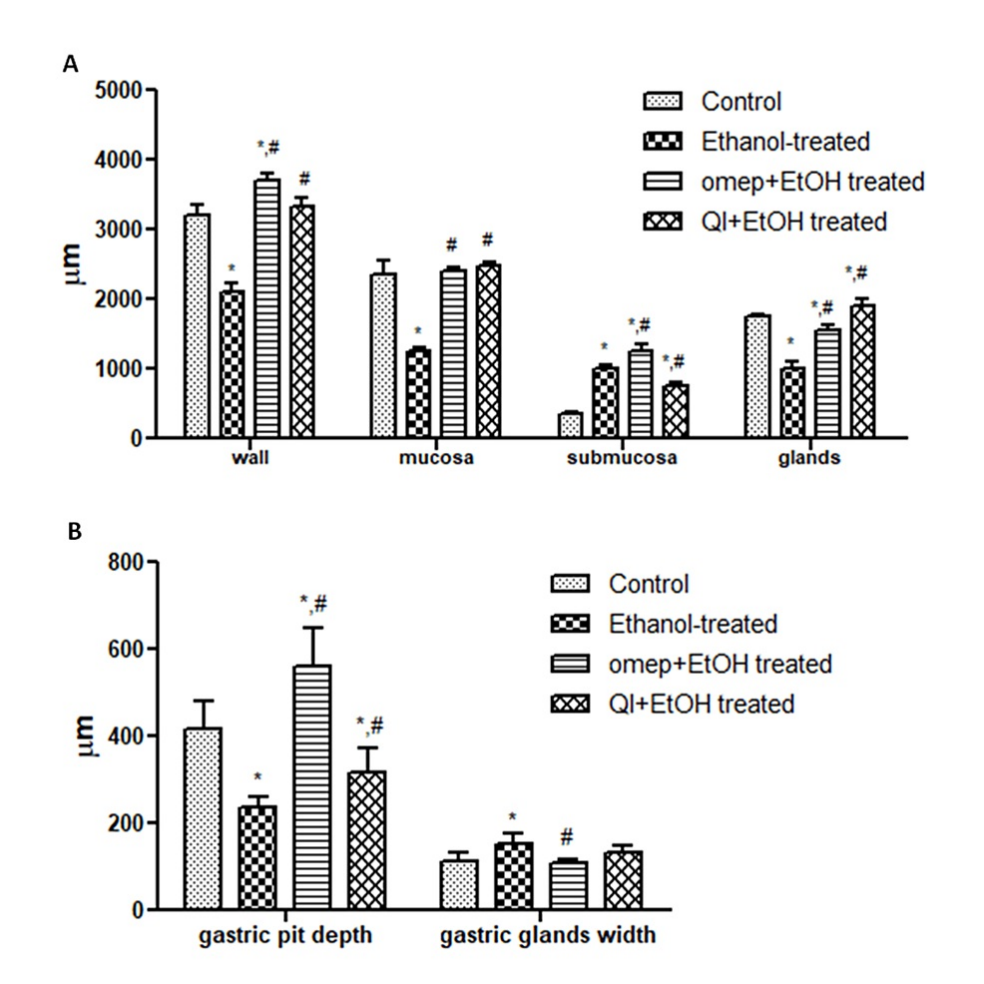
Effect of different treatments on some gastric morphological features. Ethanol treatment decreased the thickness of the gastric wall, gastric mucosa, and glands, as well as the gastric pit depth, increased submucosal thickness, and gastric gland depth. QIGE and omeprazole pre-treatment significantly retrieved the effects of ethanol treatments. Data are presented as mean ± SEM; n = 5 per group. * denotes a significant difference from healthy control, and # denotes a significant difference from ethanol-treated animals (p < 0.05). QIGE: Quercus infectoria gall extract, EtOH: ethanol, omep: omeprazole, SEM: standard error of the mean

The measurement of the thickness/length of various histological features in the gastric tissues to quantitatively compare the effect of the different treatments is shown in Figure 7. As can be seen, ethanol treatment significantly decreased the thickness of the gastric wall, gastric mucosa, and gastric glands (Figure 7A, p < 0.05). It also negatively affected gastric pit depth and gastric glands width (Figure 7B, p < 0.05), but it increased the thickness of gastric submucosa (Figure 7A, p < 0.05). Pre-treatment with omeprazole or QIGE significantly retrieved thickness of the previously mentioned histological features compared to ethanol-treated animals (gastric wall, gastric mucosa, and gastric glands; p < 0.05) in addition to improving gastric pits depth and gastric glands width and reducing the thickness of gastric submucosa (p < 0.05). This indicates the positive and protective effects of QIEG on gastric tissues.

## Discussion

Gastritis is an inflammatory condition that affects a huge population all over the world and can be attributed to various factors including *H. pylori*, NSAID administration, and chronic ethanol consumption as common risk factors. Although gastritis can be asymptomatic, untreated cases can progress into chronic atrophic gastritis, gastric ulcers, and gastric carcinoma, a prognosis that could be prevented by early intervention [16]. Several studies have demonstrated the crucial and central role of oxidative stress and reactive oxygen species in the pathogenesis of gastritis induced by various effectors, including NSAIDs and ethanol [16,17].

The first line of mechanical defense in the gastric epithelium is the mucosal membrane and the mucus it produces, which protect against the damage that could be induced by the diffusion of digestive enzymes like pepsin into the stomach wall. However, some irritants like ethanol or stress negatively impact the protective capacity of the mucosal membrane and increase its permeability [18].

Centuries ago, several herbal and natural drugs were used in folk medicine for the treatment and alleviation of gastritis and gastric ulceration. These herbal remedies were reported in a large sum of studies to function either by preventing gastric mucosal damage, supporting endogenous defense mechanisms, or modifying the secretory function of the stomach. It is to be noted that reducing the acidity of the gastric juice, e.g., via proton pump inhibitors, is one of the most effective interventions to manage gastritis and gastric ulcers; therefore, natural products that function by inhibiting the proton pump and elevating gastric pH have been reported to effectively ameliorate gastritis [19].

QI galls are reported to be rich in multiple bioactive phytochemicals, such as gallotannins that represent 50-70% of the gall content of active compounds. One important effect of tannins is their ability to interact with proteins in the superficial layers of biomembranes (e.g., mucosal lining), decreasing their permeability, and hence improving their resistance to various insults [20]. This explains the multiple therapeutic effects of QI galls in many disorders that include damage to protective lining membranes.

Interestingly, some studies reported a proton pump inhibitory effect for extracts obtained from other *Quercus* species’ leaves (*Quercus suber* and *Quercus coccifera*), which have a very similar phytochemical composition to QI galls suggesting a similar effect to QI galls [21].

In the current study, we investigated the potential protective effect of QIGE against ethanol-induced gastritis. Based on our findings, a single dose of ethanol (80%) induced serious damage to the stomach tissue on the macroscopical and microscopical levels and alteration of gastric pH and total acidity. Using QIGE as a prophylaxis for one week effectively ameliorated histopathological changes and collagen deposition in gastric submucosa and decreased gastric acidity.

One of the fundamental players in gastric injury is the hydrogen ion concentration, i.e., stomach pH, as mentioned by earlier studies and further confirmed by the efficacy of proton pump inhibitors, such as omeprazole, in treating gastritis and gastric injury [22]. This piece of information along with the elevated pH of gastric content in QIGE treated animals may, in part, explain the positive effect of the extract in the tested gastritis model.

As previously mentioned, ethanol-induced gastritis is mediated in the first place by the multifactorial increased production of ROS and oxidative stress associated with ethanol administration in addition to the direct effect of ethanol after penetrating gastric mucosa resulting in submucosal edema, hemorrhagic bands and ulcers, and loss of mucosal protection. Increased production of ROS induces lipid peroxidation and depletes endogenous antioxidants, e.g., GSH and SOD in stomach tissues, and provokes an inflammatory response as well. The direct association between oxidative stress and gastric injury explains why many antioxidant-rich herbal and natural products have proven their potential gastroprotective effects [23].

The inflammatory response induced by oxidative stress and ROS is characterized by increased macrophages and neutrophil infiltration to the site where inflammation takes place. Upon activation, these infiltrated cells start producing ROS in addition to the overexpression and release of a variety of pro-inflammatory cytokines, such as IL-6 and TNF-α, which in turn attract more immune cells to the gastric tissue, resulting in augmentation of the inflammatory response and initiating gastritis [24]. Interfering with the early events of oxidative stress is expected to hinder the subsequent events and break the vicious sequence of oxidative stress-inflammation-tissue damage [23].

In the current study, pre-treatment with QIGE effectively alleviated ethanol-induced submucosal edema and collagen deposition, which are considered as main events in gastritis pathology. This can be attributed to the previously reported antioxidant and anti-inflammatory effects of gall extract in different pathological animal models [11,12,25]. These effects of QIGE aid in counteracting ethanol-mediated oxidative stress and hence preventing the subsequent gastric edema and damage.

It is to be noted that despite the promising results in the prophylactic model, the study has some limitations, as it did not provide answers to some questions. One of them is to investigate the curative role of QIGE in animals already suffering from acute and chronic gastric ulcers. In addition, the molecular events responsible for the observed effects still need to be elucidated to provide more understanding of the mechanism of the QIGE action.

## Conclusions

The results presented in this study suggest a potential gastroprotective effect for QIGE in an ethanol-induced gastritis model. It mediates that this protective effect via QIGE exerts a direct protective effect on the mucus membranes by decreasing their permeability, improving their integrity, and increasing gastric pH, which together strengthen the gastric barrier and protect it against direct ethanol insults. In addition, it acts by ameliorating the oxidative stress status due to the already known strong antioxidant potential of the extract. Its ability to interrupt the oxidative stress-inflammation circle enables it to mitigate the direct and indirect destructive effects of ethanol. This is the first study that provides preliminary evidence for the protective effect of QIGE against gastritis. It provides a possible safe and effective solution for gastritis management as a natural product that has been safely used for decades in various inflammatory conditions. However, further experiments are needed to understand the exact mechanism of such an effect on the molecular level.
